# Supplementary material for: Patients’ experience of suffering a distal radius fracture with long-lasting impairment—a qualitative study
Source: PLoS One. 2024 Nov 26;19(11):e0311371. doi: 10.1371/journal.pone.0311371 (PMC11594481; doi:10.1371/journal.pone.0311371)
Supplement: S1 Appendix — (DOCX) [file pone.0311371.s001.docx]

Demographics of the participants in a qualitative study of the experience of a distal radius fracture

| Participant number (PN) | Gender | Age | Included from | Treatment | Fractured side  Injury to dominant hand | Radiographic feature at time for interview  -*Dorsal tilt from 0°(deg)*  *-Radial inclination (deg)*  *-Ulnar variance(mm)* | PROM-score  *-PRWE*  *-DASH*  *-EQ-5D*  *-HADS-A; HADS-D* |
| --- | --- | --- | --- | --- | --- | --- | --- |
| 001 | female | 68 | rehab | Non-operative treatment | Right  yes | 11  22  3 | 40  22  0.73  9; 3 |
| 002 | female | 63 | rehab | Surgery with volar plate | Right  yeas | 0  24  -2 | 55  51  0.62  7; 5 |
| 003 | female | 55 | rehab | Surgery with volar plate | Left  No | 0  22  -2 | 15  6  0.73  6; 1 |
| 004 | female | 60 | rehab | Surgery with volar plate | Left  No | -10  24  0 | NA |
| 005 | female | 56 | rehab | Surgery with volar plate | Left  No | -10  20  1 | 43  28  0.80  1; 1 |
| 006 | female | 33 | rehab | Surgery with volar plate | Right  Yes | -10  17  1 | 17  14  0.80  3; 5 |
| 007 | female | 79 | rehab | Non-operative treatment | Left  No | 18  17  0 | 44  47  0.80  5; 10 |
| 008 | male | 61 | SFR | Surgery with volar plate | Right  Yes | -5  26  0 | 25  20  0.12  9; 5 |
| 009 | female | 48 | Plate extractions | Surgery with volar plate | Right  Yes | -5  21  0 | 20  12  0.80  13; 10 |
| 010 | female | 72 | clinic | Surgery with volar plate | Left  No | -14  19  0 | 0  0  1  0; 1 |
| 011 | female | 51 | clinic | Non-operative treatment | Right  Yes | 3  18  -4 | NA |
| 012 | female | 54 | clinic | Surgery percutaneous pinning | Right  Yes | -12  25  -2 | 26  16  0.73  6; 4 |
| 013 | female | 81 | Referred by orthopedic surgeon | Surgery external fixation | Right  Yes | -5  23  0 | 69  53  0.0.69  6; 7 |
| 014 | female | 77 | Referred by orthopedic surgeon | Surgery with volar plate | Right  Yes | -3  17  2 | 62  63  0.73  9; 5 |
| 015 | female | 59 | clinic | Surgery with volar plate | Left  No | -8  15  -2 | NA |
| 016 | female | 55 | clinic | Surgery with volar plate | Right  Yes | -5  20  -4 | 0  1  1  3; 1 |
| 017 | male | 40 | clinic | Surgery with volar plate | Left  No | 2  26  -1 | 26  20  0.73  6; 4 |

| DASH | Disability of the Arm Shoulder and Hand |
| --- | --- |
| HAD-A | Hospital Anxiety and Depression scale - Anxiety |
| HAD-D | Hospital Anxiety and Depression scale - Depression |
| EQ-5D | Euroqol Group-5 Dimensions |
| PRWE | Patient Rated Wrist Evaluation |
| PROM | Patient Reported Outcome Measure |
