# Supplementary material for: Patients’ experience of suffering a distal radius fracture with long-lasting impairment—a qualitative study
Source: PLoS One. 2024 Nov 26;19(11):e0311371. doi: 10.1371/journal.pone.0311371 (PMC11594481; doi:10.1371/journal.pone.0311371)
Supplement: Attachment — Submitted filename: Letter including Point by point reply.docx [file pone.0311371.s002.docx]

Dear Editor,

We would like to appeal the decision of rejection since there seems to be a misunderstanding about the method used in the study. The submitted paper is a qualitative study where statistical analysis should not be used. According to your submission guidelines you consider qualitative research for publication. In the rejection all comment were based upon quantitative methods that don not apply to qualitativt research.

The quantitative research is important and what we use confirm or reject hypothesis but the qualitative research bring something different to the field. It brings another awareness of the patients experience and can generate new hypotheses that could be tested quantitively later. In distal radius fracture research qualitative studies are rare and we think this one is important to highlight the patients’ experience. It is, after all, all about the patient.

Below is a response to the concern from the Academic Editor on which the decision was based.

*1.Eventhough the merit of the study is interesting, it is based on the individual patient perception, which would benefit from larger sample and a proper statistical analysis.*

Since it is a qualitative interview study the small sample size is common and often necessary to be able to properly analyze the content, here using content analysis. A larger sample size would not necessarily add much more to this study since the data was considered rich enough to answer the research question. In this method no statistical methods would be proper to use. It could though, be interesting to use results from this study and design a quantitative study and to perform hypothesis testing in the future.

*2. Patient selection criteria is missing and the mixture of surgically and conservative treated patients bias your sample.*

We are sorry if the patient selection criteria were not as clearly described as you would like, we could further emphasis that we interviewed patients who previously had had a distal radius fracture and not perceived a satisfactory recovery. The mix of treatments was purposeful to enrich the material. Bias will always be present in qualitative studies and the results are discussed and must be interpreted with that in mind, we do not believe that the mix of treatments inhibits the interpretation of the results but rather enriches the picture with different experiences.

The original uploaded manuscript has been updated to further cohere to the coreq-checklist.

Kind regards

Hanna Südow and co-writers.
