# Supplementary material for: Patients’ experience of suffering a distal radius fracture with long-lasting impairment—a qualitative study
Source: PLoS One. 2024 Nov 26;19(11):e0311371. doi: 10.1371/journal.pone.0311371 (PMC11594481; doi:10.1371/journal.pone.0311371)
Supplement: Attachment — Submitted filename: Response to Reviewers.docx [file pone.0311371.s003.docx]

Dear Editor and reviewers!

I would like to sincerely thank you for the time and labor you have been putting into reviewing our manuscript, your knowledge.

The answers from the authors are highlighted in yellow.

Once again, thank you!

Best regards

Hanna Südow and co-authors.

1. Please ensure that your manuscript meets PLOS ONE's style requirements, including those for file naming. The PLOS ONE style templates can be found at
<https://journals.plos.org/plosone/s/file?id=wjVg/PLOSOne_formatting_sample_main_body.pdf> and
<https://journals.plos.org/plosone/s/file?id=ba62/PLOSOne_formatting_sample_title_authors_affiliations.pdf>

I apologize for not being coherent PLOS ONES’s style requirement and have made changes in the layout according to the style template.

2. Thank you for stating the following financial disclosure:
"Open access funding provided by Karolinska Institute. This study was funded by the Swedish Research Council and the Regional Agreement on Medical Training and Clinical Research between the Stockholm County Council and Karolinska Institute (ALF). CMN was supported by Region Stockholm (clinical research appointment)"

Please state what role the funders took in the study. If the funders had no role, please state: ""The funders had no role in study design, data collection and analysis, decision to publish, or preparation of the manuscript.""
If this statement is not correct you must amend it as needed.
Please include this amended Role of Funder statement in your cover letter; we will change the online submission form on your behalf.

The funders had no role in study design, data collection and analysis, decision to publish, or preparation of the manuscript.

3. Thank you for stating the following in your Competing Interests section:
"NO authors have competing interests"

Please complete your Competing Interests on the online submission form to state any Competing Interests. If you have no competing interests, please state ""The authors have declared that no competing interests exist."", as detailed online in our guide for authors at <http://journals.plos.org/plosone/s/submit-now>
This information should be included in your cover letter; we will change the online submission form on your behalf.
The authors have declared that no competing interests exist.

4. Please include captions for your Supporting Information files at the end of your manuscript, and update any in-text citations to match accordingly. Please see our Supporting Information guidelines for more information: <http://journals.plos.org/plosone/s/supporting-information>.

A caption is added

5. We note that you have indicated that there are restrictions to data sharing for this study. For studies involving human research participant data or other sensitive data, we encourage authors to share de-identified or anonymized data. However, when data cannot be publicly shared for ethical reasons, we allow authors to make their data sets available upon request. For information on unacceptable data access restrictions, please see <http://journals.plos.org/plosone/s/data-availability#loc-unacceptable-data-access-restrictions>.

Before we proceed with your manuscript, please address the following prompts:

a) If there are ethical or legal restrictions on sharing a de-identified data set, please explain them in detail (e.g., data contain potentially identifying or sensitive patient information, data are owned by a third-party organization, etc.) and who has imposed them (e.g., a Research Ethics Committee or Institutional Review Board, etc.). Please also provide contact information for a data access committee, ethics committee, or other institutional body to which data requests may be sent.

b) If there are no restrictions, please upload the minimal anonymized data set necessary to replicate your study findings to a stable, public repository and provide us with the relevant URLs, DOIs, or accession numbers. Please see <http://www.bmj.com/content/340/bmj.c181.long> for guidelines on how to de-identify and prepare clinical data for publication. For a list of recommended repositories, please see <https://journals.plos.org/plosone/s/recommended-repositories>. You also have the option of uploading the data as Supporting Information files, but we would recommend depositing data directly to a data repository if possible.

Please update your Data Availability statement in the submission form accordingly.

The datasets generated and analysed during the current study are not publicly available due to the Swedish ethical review regulation. Data are available upon reasonable request. Inquiries for data access should be sent to Institutionen för Klinisk forskning och utbildning Södersjukhuset , Karolinska Institutet, Sjukhusbacken 10,  118 83 Stockholm or contact the corresponding author, Hanna Südow, hanna.sudow@ki.se , who will then contact the Swedish Ethical Review Authority for permission to share the data.

Please review your reference list to ensure that it is complete and correct. If you have cited papers that have been retracted, please include the rationale for doing so in the manuscript text, or remove these references and replace them with relevant current references. Any changes to the reference list should be mentioned in the rebuttal letter that accompanies your revised manuscript. If you need to cite a retracted article, indicate the article’s retracted status in the References list and also include a citation and full reference for the retraction notice.
The reference list is reviewed and I cannot find that any cited articles has been retracted.

**Comments to the Author**

1. If the authors have adequately addressed your comments raised in a previous round of review and you feel that this manuscript is now acceptable for publication, you may indicate that here to bypass the “Comments to the Author” section, enter your conflict of interest statement in the “Confidential to Editor” section, and submit your "Accept" recommendation.

Reviewer #1: (No Response)

Reviewer #2: All comments have been addressed

2. Is the manuscript technically sound, and do the data support the conclusions?

The manuscript must describe a technically sound piece of scientific research with data that supports the conclusions. Experiments must have been conducted rigorously, with appropriate controls, replication, and sample sizes. The conclusions must be drawn appropriately based on the data presented.

Reviewer #1: Partly

Reviewer #2: Yes

3. Has the statistical analysis been performed appropriately and rigorously?

Reviewer #1: N/A

Reviewer #2: N/A

4. Have the authors made all data underlying the findings in their manuscript fully available?

The [PLOS Data policy](https://eur01.safelinks.protection.outlook.com/?url=http%3A%2F%2Fc05y1x9s.r.us-east-2.awstrack.me%2FL0%2Fhttp%3A%252F%252Fwww.plosone.org%252Fstatic%252Fpolicies.action%2523sharing%2F1%2F010f01912f66e10f-cf6d1fd4-de60-4fcf-8298-7a4bda50f39a-000000%2FuNB4SbOmvslYWiuv2BvVvFcO9Xo%3D171&data=05%7C02%7Channa.sudow%40ki.se%7C6dec928de1554537a40208dcb7419b58%7Cbff7eef1cf4b4f32be3da1dda043c05d%7C0%7C0%7C638586741271320851%7CUnknown%7CTWFpbGZsb3d8eyJWIjoiMC4wLjAwMDAiLCJQIjoiV2luMzIiLCJBTiI6Ik1haWwiLCJXVCI6Mn0%3D%7C0%7C%7C%7C&sdata=dYGCFFSfgiVMDdC0xmrT%2FSgbIB%2Bc8IDB5l7Q9as3SoE%3D&reserved=0) requires authors to make all data underlying the findings described in their manuscript fully available without restriction, with rare exception (please refer to the Data Availability Statement in the manuscript PDF file). The data should be provided as part of the manuscript or its supporting information, or deposited to a public repository. For example, in addition to summary statistics, the data points behind means, medians and variance measures should be available. If there are restrictions on publicly sharing data—e.g. participant privacy or use of data from a third party—those must be specified.

Reviewer #1: Yes

Reviewer #2: Yes

5. Is the manuscript presented in an intelligible fashion and written in standard English?

PLOS ONE does not copyedit accepted manuscripts, so the language in submitted articles must be clear, correct, and unambiguous. Any typographical or grammatical errors should be corrected at revision, so please note any specific errors here.

Reviewer #1: Yes

Reviewer #2: Yes

6. Review Comments to the Author

Please use the space provided to explain your answers to the questions above. You may also include additional comments for the author, including concerns about dual publication, research ethics, or publication ethics. (Please upload your review as an attachment if it exceeds 20,000 characters)

Reviewer #1: Although this is an interesting qualitative study, the included numbers are small.
It would have been benificial, if some scores would have been included, like the DASH score, SF 36 and the EQ-5D-5L score. Please add a comment in your conclusion that this is a further limitation of the study and explain why scores these scores were not performed.

This is a good point and we have been spending a lot of time discussion whether we should include any quantitative data in the study or not.

When we started the study, we collected both DASH, PRWE, EQ-5D HAD. Any proper statistic, in this setting with the purposeful sampling, would however per definition be biased. We will provide the PROM and radiograph as demographics as an appendix for anyone to learn more about the material, line 162.

Reviewer #2: 1) General comment
The authors would like to show in their qualitative study protocoll “Patients` experience of suffering a distal radius fracture with long-lasting impairment” that the measurement of a such impairment after a distal radius fracture is very difficult. And the experience as being patients, the level of knowledge and their personal circumstances play a additional role in understanding how the participants experienced their recovery.
In this qualitative study the authors show how the view of those affected is and put this in the center of the research interest. They collect the data and use it dynamically to providing the hypothesis and theories. Their data explore the directions of patients with distal radius fractures which are operated or conservatively treated. The interviews and qualitative content analysis underline their conclusions by means of their results.
The study design is very well thought out or worked out for the questions mentioned. However, it is worth discussing about the persistent impairment of distal radius fracture without pathological radiological signs, being a patient and their personal circumstances. It is very interesting how affects these facts the recovery process of the patients.
There are a lot of quantitative studies describing the correlation between radiological findings and clinical outcomes. But it does exist only a few single studies in which the personal circumstances or being a patient influence the recovery process of any fractures.
However, it is necessary to investigate these correlation by interviewing patient to explore their personal needs and experiences. And further study protocols could lead to a better understanding of these connections. Based on this conclusions there is a possibility to a personalized treatment algorhythm. And maybe it could to a more optimal outcome of such patients with a distal radius fracture with a persistent clinical impairment
Finally it would save some economical costs if these patient could get earlier to work, to reach faster their independence and to avoid additional healthcare.

2) Specific suggestions
The qualitative study mentioned above is well designed and will be appropriate for the intended research question and outcome results. Their results by interviews underline the conclusion that patients suffering from considerable impairment could be difficult detected after a distal radius fracture even if the radiological outcome is successful. The three main categories are specifically worked out by the authors. However, persistent impairment and how it affects life, their experience as being patients and their personal circumstances play an important role in how the participants perceice their recovery.

7. PLOS authors have the option to publish the peer review history of their article ([what does this mean?](https://eur01.safelinks.protection.outlook.com/?url=https%3A%2F%2Fc05y1x9s.r.us-east-2.awstrack.me%2FL0%2Fhttps%3A%252F%252Fjournals.plos.org%252Fplosone%252Fs%252Feditorial-and-peer-review-process%2523loc-peer-review-history%2F1%2F010f01912f66e10f-cf6d1fd4-de60-4fcf-8298-7a4bda50f39a-000000%2F4BxD_MW4jmrCdT54QDj_cByUHBw%3D171&data=05%7C02%7Channa.sudow%40ki.se%7C6dec928de1554537a40208dcb7419b58%7Cbff7eef1cf4b4f32be3da1dda043c05d%7C0%7C0%7C638586741271327532%7CUnknown%7CTWFpbGZsb3d8eyJWIjoiMC4wLjAwMDAiLCJQIjoiV2luMzIiLCJBTiI6Ik1haWwiLCJXVCI6Mn0%3D%7C0%7C%7C%7C&sdata=5OuK4h3o1rJUuzGKeeuLi%2FTihJiGQhOu2XAB0LExZxM%3D&reserved=0)). If published, this will include your full peer review and any attached files.


If you choose “no”, your identity will remain anonymous but your review may still be made public.


**Do you want your identity to be public for this peer review?** For information about this choice, including consent withdrawal, please see our [Privacy Policy](https://eur01.safelinks.protection.outlook.com/?url=https%3A%2F%2Fc05y1x9s.r.us-east-2.awstrack.me%2FL0%2Fhttps%3A%252F%252Fwww.plos.org%252Fprivacy-policy%2F1%2F010f01912f66e10f-cf6d1fd4-de60-4fcf-8298-7a4bda50f39a-000000%2FEIOC_f4QxGdPnL1Bxpy8DS5FZo0%3D171&data=05%7C02%7Channa.sudow%40ki.se%7C6dec928de1554537a40208dcb7419b58%7Cbff7eef1cf4b4f32be3da1dda043c05d%7C0%7C0%7C638586741271335277%7CUnknown%7CTWFpbGZsb3d8eyJWIjoiMC4wLjAwMDAiLCJQIjoiV2luMzIiLCJBTiI6Ik1haWwiLCJXVCI6Mn0%3D%7C0%7C%7C%7C&sdata=PPPfaQJ%2BNxC4U1DvcV7J5RBKrVTCfOQ%2BZ%2Fw%2FOafbDtg%3D&reserved=0).

Reviewer #1: No

Reviewer #2: No
